# Mediating Effect of Self-Efficacy on the Relationship Between Medication Literacy and Medication Adherence Among Patients With Hypertension

**DOI:** 10.3389/fphar.2020.569092

**Published:** 2020-12-07

**Authors:** Zhiying Shen, Shuangjiao Shi, Siqing Ding, Zhuqing Zhong

**Affiliations:** ^1^ Department of Hematology, Third Xiangya Hospital, Central South University, Changsha, China; ^2^ Department of Nursing, Third Xiangya Hospital, Central South University, Changsha, China; ^3^ Clinical Nursing Safety Management Reasearch Center of Central South University, Third Xiangya Hospital, Central South University, Changsha, China

**Keywords:** self-efficacy, medication literacy, medication adherence, hypertension, mediating effect

## Abstract

**Background:** Studies have reported that medication literacy had a positive effect on medication adherence in patients with hypertension. However, little is known about the mechanism underlying this relationship in patients with hypertension.

**Objective:** The purpose of this study was to investigate the mediating effect of self-efficacy between medication literacy and medication adherence.

**Methods:** A total of 790 patients with hypertension were investigated using the Chinese Medication Literacy Scale for Hypertensive Patients (C-MLSHP), the Morisky Medication Adherence Scale-8 (MMAS-8) and the Medication Adherence Self-efficacy Scale-Revision (MASES-R). Hierarchical regression and the bootstrap approach were used to analyze the mediating effect of self-efficacy on the relationship between medication literacy and medication adherence.

**Results:** A total of 60.9% of hypertensive patients were low adherent to their antihypertensive drug regimens. Self‐efficacy had a significant positive correlation with medication literacy (*r*= 0.408, *p* < 0.001) and medication adherence (*r* = 0.591, *p* < 0.001). Self-efficacy accounts for 28.7% of the total mediating effect on the relationship between medication literacy and adherence to antihypertensive regimens for hypertensive patients.

**Conclusion:** More than half of the hypertensive patients in the study were low adherent to antihypertensive regimens. Self-efficacy had a partial significant mediating effect on the relationship between medication literacy and medication adherence. Therefore, it was suggested that hypertensive patients’ medication adherence might be improved and driven by increasing self-efficacy. Targeted interventions to improve patients’ self-efficacy should be developed and implemented. In addition, health care providers should also be aware of the importance of medication literacy assessment and promotion in patients with hypertension.

## Introduction

Hypertension has caused great damage to human health and consumed a large amount of medical resources worldwide, it is a leading problem in global public health management and promotion (Irazola et al., 2016). Poor blood pressure control can eventually lead to various complications and comorbidities, such as heart diseases, stroke and kidney failure, as well as increasing premature mortality and disability, which has contributed to high costs in dealing with these medical outcomes (World Health Organization, 2013). Strict early control of blood pressure has been shown to be beneficial in extending life expectancy in hypertensive patients (Vaduganathan et al., 2020). According to the latest data released by the “Report on Disease of Cardiovascular in China 2019”, 330 million people in China have been suffering from cardiovascular diseases, among which 245 million patients have been diagnosed with hypertension (Hu et al., 2020).

Lifestyle change and antihypertensive medication are considered the most prevalent and agreed-upon guidelines for the effective management of hypertension (Weber et al., 2014). Adherence is recognized as a key factor in the effectiveness of antihypertensive medication treatment. However, patients’ poor adherence to antihypertensive regimens is a prevalent problem that has limited the efficacy of antihypertensive drugs and leads to suboptimal blood pressure control (Abegaz et al., 2017; Hamdidouche et al., 2017). A review analyzed 24 studies and found that approximately 31% of cases of resistant hypertension may be attributed to poor adherence to the medication regimens (Hamdidouche et al., 2017). Another meta-analysis of 28 studies showed that among 12,603 hypertensive patients, 45.2% were nonadherent to antihypertensive medication, and 83.7% of patients with nonadherence were found to have uncontrolled blood pressure (Abegaz et al., 2017). In addition, nonadherence to antihypertensive drugs in patients with hypertension was significantly associated with a higher risk of stroke, coronary heart disease, and chronic heart failure (Shin et al., 2013; Lee et al., 2017; Jinkwon et al., 2018). Therefore, nonadherence to medication regimens continues to be a prevalent barrier to achieving optimal blood pressure and health outcomes in patients with hypertension.

For hypertensive patients in low- and middle- income countries, the rates of non-adherence to hypertensives regimen were up to from 45.2 to 66.7% (Abegaz et al., 2017; Nielsen et al., 2017; Rampamba et al., 2018). Only 6.2% of hypertensive patients had high adherence to their medication regimens in Saudi Arabia (Fatani et al., 2019). High rates of poor adherence to medication regimens for Chinese hypertensive patients were also found in several studies (63.6–78.7%) (Hou et al., 2016; Pan et al., 2017; Shi et al., 2019). In addition, different kinds of associated factors of poor or non-adherence have also been confirmed in lots of recent studies. For example, socio-demographic factors including gender, age, education level, occupational status, or even race; (Abegaz et al., 2017; Lee et al., 2017; Fatani et al., 2019) socio-economic status including annual income and medical insurance; (Boima et al., 2015; Nielsen et al., 2017) clinical characteristics for patients including family disease history, number of prescribed drugs, comorbidity, and duration of hypertension (Choi et al., 2018; Uchmanowicz et al., 2019). Most importantly, psychosocial factors also exert significant influence on medication adherence, including depressed emotion, perceived severity of disease, self-rated health, perceived symptoms, and self-efficacy (Al-Noumani et al., 2018; Asgari et al., 2019).

Previous studies have shown that hypertension patients with higher health literacy also have higher adherence to medication(Mcnaughton et al., 2014; Lor et al., 2019) People with low levels of health literacy were more likely to misinterpret information on drug labels and less likely to participate in drug decision-making and actively communicate drug information with doctors (Aboumatar et al., 2013; AbuAlreesh and Alburikan, 2019). In addition, medication literacy is health literacy in the context of medication use (Ngoh, 2009; Peiravian et al., 2014). The definition of medication literacy is the degree to which individuals can obtain, comprehend, communicate, calculate, and process patient-specific information about their medication to make informed medication and health decisions in order to safely and effectively use their medications regardless of the mode by which the content is delivered (e.g., written, oral, and visual) (Pouliot et al., 2018). Four core elements of medication literacy include knowledge, attitude, skill and behavior. Each domain is essential and critical for processing medication information and correct medication use (Zheng et al., 2017; Shi et al., 2019) In the process of disease self-management, medication literacy, to a certain extent, determines how well patients can manage their medication regimens correctly and tailor their medication behaviors. Medication literacy can be used as a significant predictor of correct medication use (Zheng et al., 2015). In the study of Shi et al. (Shi et al., 2019) medication literacy was found to be a positive independent predictor of medication adherence for hypertensive patients. However, the specific mechanism mediating the relationship between hypertensive patients’ medication literacy and their adherence to medication regimens remains unclear and needs to be further studied.

Self-efficacy refers to the individual's confidence to make use of his or her own ability to achieve a certain goal, which can determine the individual's choices, persistence and effort toward the task. It also affects the individual's way of thinking and feeling in the process of executing the task (Bandura et al., 1999). Previous studies have shown that self-efficacy was one of the determinants of medication adherence in patients with chronic diseases (Daniali et al., 2017; Huang et al., 2018). Patients with high levels of self-efficacy had greater confidence that they would be willing to take antihypertensive drugs as prescribed on different occasions (Schoenthaler et al., 2016; Yang et al., 2016). In other words, individuals with higher self-efficacy level have significantly increased chances of adhering to medication regimens (Elder et al., 2012; Warren-Findlow et al., 2012; Alhalaiqa et al., 2013). Moreover, self-efficacy can not only directly affect patients’ adherence to medication but also mediates the relationship between medication adherence and a variety of psychosocial factors, such as health literacy, depression, and weight discrimination (Richardson et al., 2014; Son and Won, 2017; Huang et al., 2018; Huang et al., 2018). Considering that medication literacy is health literacy in the context of medication use, we can reasonably assume that self-efficacy may be an important mediating factor between medication literacy and medication adherence.

To our knowledge, there have been few studies exploring the role of self-efficacy in mediating medication literacy and medication adherence in patients with hypertension. Knowledge about the specific role of self-efficacy in the relationship between medication literacy and medication adherence may help to develop effective interventions to promote hypertensive patients’ adherence to their medication regimens and improve health outcomes. Thus, the purpose of this study was to investigate the mediating effect of self-efficacy on the relationship between medication literacy and medication adherence.

## Methods

### Study Design

This was a cross-sectional study and was conducted at five general hospitals and three community healthcare services in a southern province of China from March 2018 to August 2018. Purposive sampling method was used in this study. One questionnaire with three scales were administered to hypertensive patients in the outpatient department face to face. For completing three different evaluating scales along with the characteristic information questionnaires, it took about 20 min for each patient. All the patients who participated in the study signed the informed consent in person.

### Participants and Procedures

Patients were included if they 1) were aged 18 years or older; 2) had been diagnosed with hypertension by a cardiologist; 3) had been on antihypertensive treatment for at least 2 weeks; 4) speak Chinese and communicated well with others; and 5) understood the purpose and process of the study and agreed to participate. Patients were excluded if they 1) had other serious diseases, such as cancer, acute myocardial infarction, cerebral hemorrhage or chronic renal failure; 2) had secondary hypertension, such as elevated blood pressure caused by chronic renal dysfunction diseases; or 3) were diagnosed as psychological or mental impairment according to International Classification of Diseases (ICD) guideline, or were on the psychotherapy treatment. Eligible hypertensive patients were invited to participate in the study. They were provided with information on the purpose and content of the study, the investigation procedures, and the principle of anonymity of this study. The questionnaires were completed after the patient signed the informed consent form. For illiterate patients, we communicated with both them and their family members, if they agree to participate in the study, then they were instructed by one of their family members to sign the informed consent forms. In the present study, 5 master’s degree students were trained to distribute and collect the questionnaires. For the illiterate participants, the researchers read the questions verbatim and recorded their answers. All questionnaires were immediately collected onsite upon completion, and collected questionnaires were checked for any missing information to ensure data integrity.

### Data Collection Tools

#### Sociodemographic and Clinical Characteristics

The following information about patients’ sociodemographic and clinical characteristics was collected using a self‐made questionnaire: age, gender, education level, annual income, duration of hypertension, number of antihypertensive drugs prescribed, and number of times antihypertensive drugs taken daily.

#### Chinese Medication Literacy Scale for Hypertensive Patients

C-MLSHP is a self-administered medication literacy measure for hypertensive patients, and it was developed by our research team (Zhong et al., 2020). This scale included 37 items on four domains of knowledge, attitude, skill, and behavior. The knowledge domain has 9 items, the attitude domain includes 8 items, the skill domain has 7 items, and the behavior domain involves 13 items. The total score for this scale ranges from 0 to 37, and higher scores indicate higher medication literacy levels. Specifically, in the knowledge and skill domains, answering right for each item scores 1, and a wrong answer for each item scores 0. Each item in the attitude and behavior domains has a 5-point Likert response, and scores of 1.0, 0.75, 0.5, 0.25, and 0 are assigned to the respective answers. In addition, 5 items in the attitude domain and 1 item in the behavior domain are scored in a reverse way.

For the C-MLSHP, 637 Chinese hypertensive patients were included for reliability and validity test. The calculated Cronbach’s α coefficient for the overall scale was 0.849, and for each domain, the Cronbach’s α coefficients ranged from 0.744 to 0.783. For the whole scale, the calculated split-half reliability was 0.893, and for each domain, it ranged from 0.793 to 0.872. The calculated test-retest reliability of the whole scale was 0.968. For each domain, the test-retest reliability coefficients ranged from 0.880 to 0.959. Therefore, good reliability of C-MLSHP was confirmed. Good content validity and acceptable construct validity of the whole scale was also confirmed. It showed a good content validity index above 0.8 for each item of this scale and for the overall scale (0.968).

#### Morisky Medication Adherence Scale-8

The MMAS-8 was originally developed by Morisky and his research team (Morisky et al., 2008). It is a concise, pragmatic and cost-effective self-administered measure, mainly used to evaluate medication adherence level. The scale includes 8 items and is confirmed to have good reliability and validity in patients with hypertension. The Cronbach’s alpha coefficient of this scale was 0.83. In this scale, yes and no are the answer options for seven items, and the last question is answered on a 5-point Likert scale. The total score on this scale ranges from 0∼8. Higher scores represent better adherence to hypertensive drugs. Morisky’s suggested cut-off point of 6 was applied: MMAS score <6 (low adherence), score =8 (high adherence), and score ≥ 6 and <8 (medium adherence). The Chinese version of the MMAS-8 (C-MMAS-8) was translated by Yan, and it was first applied in Chinese myocardial infarction patients (Yan et al., 2014). Good reliability and validity (Cronbach’s α = 0.77, pretest–posttest correlation coefficient 0.88) were identified in Chinese myocardial infarction patients (Yan et al., 2014). Every item of the Chinese version of MMAS-8 that was used in the present study has nothing different from the original English version except from the language difference.

#### Medication Adherence Self-Efficacy Scale-Revision

MASES-R is a self-administered scale with a single domain including 13 items. It was originally adapted for hypertensive African Americans by Professor Ogedegbe and his group at New York University School of Medicine (Fernandez et al., 2008). It aims to measure medication adherence self-efficacy for hypertensive patients. All the items in this scale cover a variety of circumstances hypertensive patients may encounter during the process of their everyday medication administration. Each item has a 4-point Likert response scale (0 = not sure at all, 1 = a little sure, 3 = pretty sure, 4 = fully sure). The total score for this scale is calculated as the average score of all the items, ranging from 1 to 4. A higher score indicates higher medication adherence self-efficacy. We were authorized by Professor Ogedegbe to translate the MASES-R into Chinese version and test its reliability and validity in 445 Chinese hypertensive patients. Acceptable reliability and validity were identified. Specifically, the correlation coefficients between each item and the total scale ranged from 0.660 to 0.919, and the correlations for each item ranged from 0.514–0.872. As for the validity of this scale, the I-CVI for each item was 0.83–1.00, and the S-CVI for the whole scale was 0.961. For exploratory factor analysis, the KMO value was 0.920, and Bartlett's spherical test chi-square value was 6405.74 (*p* < 0.001). The factor loading coefficient ranged from 0.640 to 0.916, and the cumulative variance contribution rate of the overall scale was 68.72%. The Cronbach's α coefficient of the scale was 0.960, and the Spearman-Brown split-half reliability was 0.927.

### Data Analysis

**FIGURE 1:**
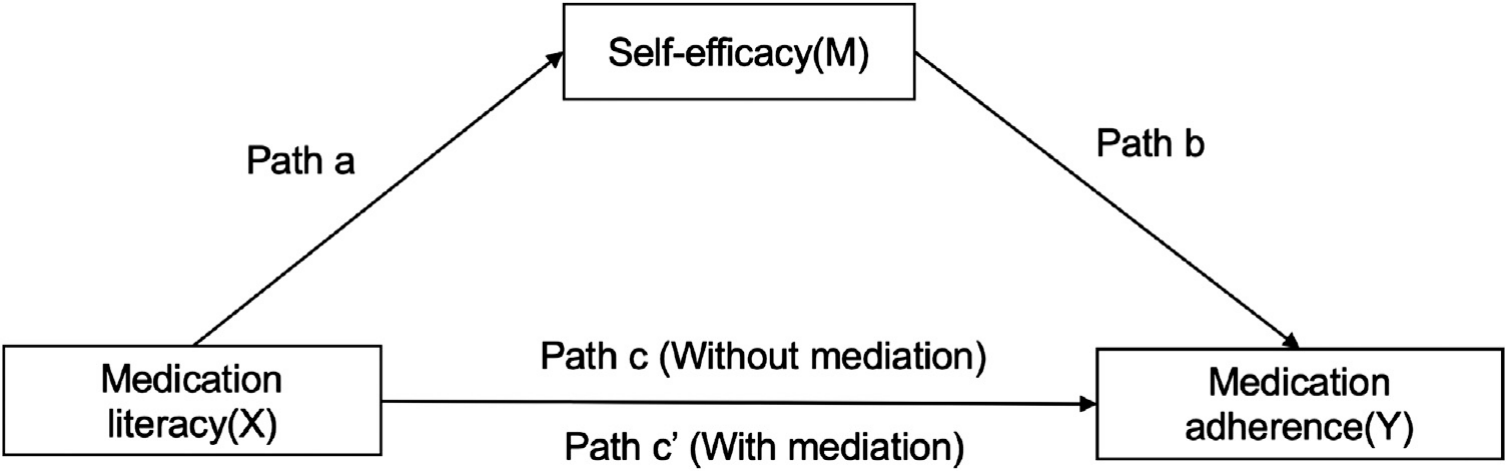
Theoretical framework of this study.

**FIGURE 2:**
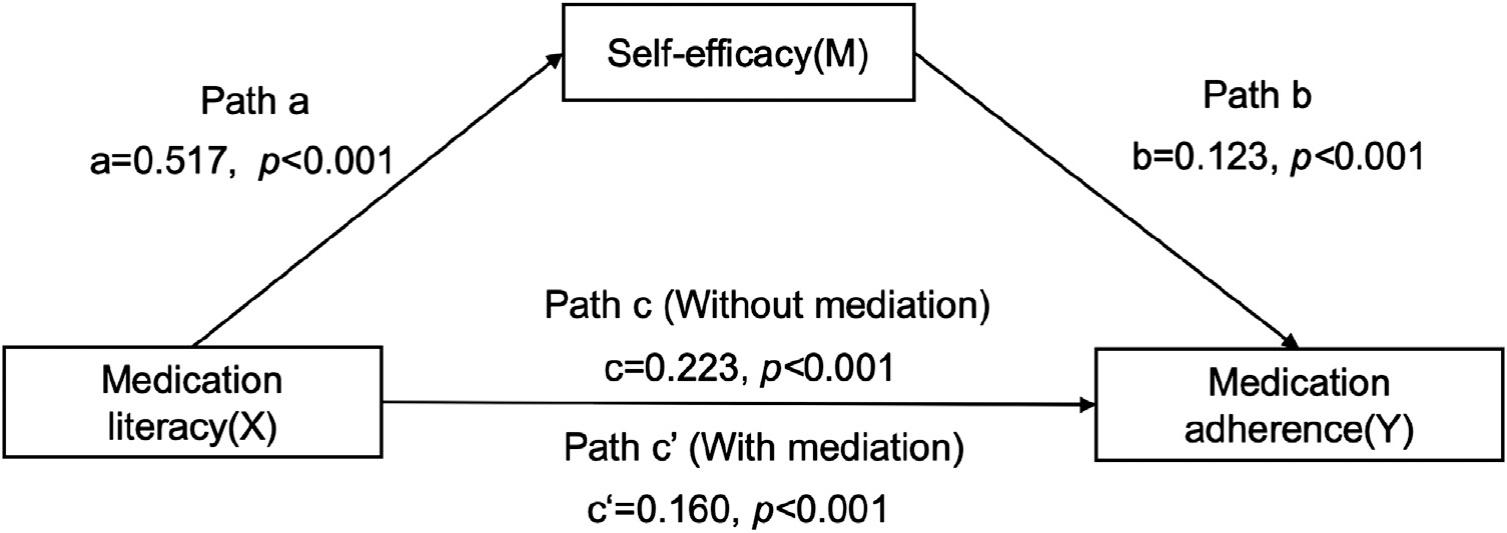
Mediating role of self‐efficacy on the relationships between. medication literacy and medication adherence.

All data were analyzed using SPSS 24.0 (IBM Corp., Armonk, NY, USA). All continuous variables with normal distribution were described in means and standard deviation (mean ± SD), and the categorical variables were summarized by numbers or percentages. The scores of medication literacy, self-efficacy, and medication adherence among hypertensive patients with different sociodemographic and clinical characteristics were compared using the independent-sample *t* test or analysis of variance. Pearson correlation analysis was used to determine the correlation among medication literacy, self-efficacy, and medication adherence. The mediating analytic framework described by Baron and Kenny (Baron and Kenny, 1986) guided the analysis plan. The capital letters X, M, and Y were used to represent medication literacy, self-efficacy, and medication adherence, respectively. Variable M was considered a mediator if 1) X significantly predicted Y directly (Path c in Figures 1, 2) X significantly predicted M (Path a in Figure 1), or 3) M significantly predicted Y after controlling for X (Path b Figure 1) (Huang et al., 2018). Path c' meant the direct effect of X on Y after controlling for M (Path c' in Figure 1). If the regression correlation coefficient of path c' was not significant, then this mediating effect of M was complete mediation. If the regression correlation coefficient of path c' was significant, then this mediating effect of M was partial mediation. The mediation effect value was calculated as a*b, and the ratio of the mediating effect with the total effect was a*b/c. The mediation effect value was tested by a bootstrap approach to verify the existence of a mediation effect (a is the regression correlation coefficient of path a; b is the regression correlation coefficient of path b; c or c' is the regression correlation coefficient of path c or path c') (Preacher and Hayes, 2004). A two-sided test was performed at a 0.05 significance level.

## Results

### Scores of Medication Literacy, Self‐Efficacy, and Medication Adherence in Hypertensive Patients

In total, 850 hypertensive patients were surveyed in this study, and 790 surveys were completed, yielding a response rate of 92.94%. Demographic and clinical characteristics, medication literacy, self‐efficacy, and medication adherence scores of the studied participants are presented in Table 1. Participants with different education levels, annual income and different number of antihypertensive drugs prescribed had significantly different scores of medication literacy, adherence to medication and self-efficacy. Age difference in patients could lead to varying medication adherence level in a significant level. Different duration of hypertension for participants had significantly different medication literacy level. In addition, seventy-two (9.1%) of the hypertensive patients had high medication adherence, 237 (30.0%) had moderate medication adherence, and 481 (60.9%) had low medication adherence. The average scores for medication literacy, self‐efficacy, and medication adherence were 23.83 ± 4.99, 3.04 ± 0.54, and 4.95 ± 2.16, respectively.

**TABLE 1:** Scores on medication literacy, self-efficacy and medication adherence of hypertensive patients of different characteristics (N = 790).

Factors	Items	N	Medication literacy	Self-efficacy	Medication adherence
Age	18–44	40	22.90 ± 5.84	2.92 ± 0.59	4.82 ± 2.43
—	45–59	329	23.56 ± 4.74	3.00 ± 0.52	4.65 ± 2.17
—	≥60	421	24.13 ± 5.09	3.08 ± 0.55	5.19 ± 2.09
*F* value	—	—	1.916	2.890	5.094
*p* value	—	—	0.148	0.056	0.003
Gender	Male	427	0.64 ± 0.14	3.03 ± 0.53	4.85 ± 2.25
—	Female	363	0.64 ± 0.13	3.05 ± 0.56	5.06 ± 2.04
*F* value	—	—	0.037	0.186	1.885
*p* value	—	—	0.847	0.666	0.170
Education level	Primary or below	233	0.59 ± 0.12	2.91 ± 0.52	4.13 ± 2.07
—	Junior middle school	213	0.65 ± 0.14	3.01 ± 0.58	4.85 ± 2.31
—	Senior high school or secondaryspecialized school	208	0.66 ± 0.12	3.11 ± 0.48	5.40 ± 1.98
—	Junior college	93	0.69 ± 0.13	3.17 ± 0.54	5.69 ± 1.79
—	Bachelor degree or above	43	0.74 ± 0.14	3.19 ± 0.62	6.13 ± 1.77
*F* value	—	—	18.301	6.938	18.296
*p* value	—	—	0.000***	0.000***	0.000***
Annual income	<10,000/year	94	0.61 ± 0.13	2.88 ± 0.60	4.06 ± 2.62
—	10,000–29,999/year	163	0.63 ± 0.15	2.94 ± 0.55	4.33 ± 2.09
—	30,000–49,999/year	241	0.62 ± 0.12	3.06 ± 0.54	5.09 ± 1.91
—	50,000–99,999/year	198	0.66 ± 0.12	3.10 ± 0.45	5.32 ± 2.00
—	≥100,000/year	94	0.72 ± 0.14	3.18 ± 0.57	5.79 ± 2.16
*F* value	—	—	11.370	5.563	13.416
*p* value	—	—	0.000***	0.000***	0.000***
Duration of hypertension	<3 years	105	0.62 ± 0.15	2.98 ± 0.54	4.79 ± 2.54
—	3–4.9 years	118	0.61 ± 0.14	3.02 ± 0.53	4.64 ± 2.10
—	5–9.9 years	288	0.65 ± 0.13	3.06 ± 0.48	4.99 ± 2.01
—	≥10 years	279	0.66 ± 0.14	3.04 ± 0.60	5.09 ± 2.17
*F* value	—	—	5.001	0.544	1.419
*p* value	—	—	0.002**	0.652	0.236
Number of antihypertensive drugs prescribed	One	629	0.65 ± 0.13	3.06 ± 0.52	5.01 ± 2.06
—	2–3 kinds	134	0.65 ± 0.14	2.95 ± 0.63	4.83 ± 2.45
—	4 or more	27	0.58 ± 0.13	2.82 ± 0.37	4.01 ± 2.74
*F* value	—	—	3.440	4.586	3.068
*p* value	—	—	0.033*	0.010*	0.047*
Number of times antihypertensive drugs taken daily	Once	632	0.64 ± 0.13	3.07 ± 0.52	4.92 ± 2.11
—	2–3 times	138	0.65 ± 0.15	2.91 ± 0.62	5.28 ± 2.39
—	4 or more	20	0.60 ± 0.12	2.87 ± 0.31	3.70 ± 1.44
*F* value	—	—	1.432	5.967	5.143
*p* value	—	—	0.240	0.003**	0.006**

Note: *p < 0.05; **p < 0.01; ***p < 0.001. The level of adherence was measured through the eight-item Morisky Medication Adherence Scale (MMAS-8). Use of the MMAS is protected by US copyright laws. Permission for use is required. A licensing agreement is available from: Donald E. Morisky, ScD, ScM, MSPH. Use of the ©MMAS is protected by US copyright and registered trademark laws. Permission for use is required. A licensing agreement is available from: Donald E. Morisky, 294 Lindura Court, Las Vegas, NV 89138-4632; dmorisky@gmail. com. The scale’s questions are available in the originally published article.

### Correlations Between Medication Literacy, self‐efficacy, and Medication Adherence

The scores for the total medication literacy scale and for each dimension were positively correlated with the score for the self‐efficacy scale at a significant level (*r* = 0.408, *p* < 0.001). The scores for the total medication literacy scale and for each dimension were also positively correlated with the score for the scale of medication adherence (*r* = 0.585, *p* < 0.001). In addition, the score for the self‐efficacy scale was significantly positively correlated with the score for medication adherence (*r* = 0.591, *p* < 0.001) (Table 2).

**TABLE 2:** Correlation between hypertensive patients’ medication literacy,self-efficacy and medication adherence.

Variables	Knowledge literacy	Attitude literacy	Skill literacy	Behavior literacy	Medication literacy	Self-efficacy	Medication adherence
Knowledge literacy	1	—	—	—	—	—	—
Attitude literacy	0.377**	1	—	—	—	—	—
Skill literacy	0.412**	0.316**	1	—	—	—	—
Behavior literacy	0.325**	0.349**	0.235**	1	—	—	—
Medication literacy	0.768**	0.643**	0.705**	0.701**	1	—	—
Self-efficacy	0.294**	0.334**	0.264**	0.285**	0.408**	1	—
Medication adherence	0.422**	0.493**	0.295**	0.478**	0.585**	0.591**	1

Note: **p < 0.01.

### Analysis of the Mediating Role of Self-Efficacy Between Medication Literacy and Medication Adherence

Figure 2 indicates the mediating role of self‐efficacy in the relationship between medication literacy and medication adherence. The results showed that after controlling for sociodemographic and clinical variables, a significant total effect of medication literacy on medication adherence was identified (Path c: c = 0.223, *t* = 17.396, *p* < 0.001). In path a, medication literacy had a positive impact on self-efficacy (Path a:a = 0.517, *t* = 10.753, *p* < 0.001). In addition, both medication literacy and self-efficacy had a positive impact on medication adherence (Path c': c' = 0.160, *t* = 13.073, *p* < 0.001; b = 0.123, *t* = 14.514, *p* < 0.001). The mediation effect value was calculated as 0.517*0.123, that is, 0.064, and the ratio of the mediating effect over the total effect was 28.7% (0.064/0.223 = 0.287). A summary of the mediating effects of self-efficacy between medication literacy and medication adherence is shown in Table 3.

**TABLE 3:** Summary of the mediating effects of self-efficacy between medication literacy and medication adherence.

Effect	Independent variables	Dependent variables	B	SE	*t*	*p* value	95%CI
Total effect(c)	X	Y	0.223	0.013	17.396	0.000***	0.198–0.248
Indirect effect(a)	X	M	0.517	0.048	10.753	0.000***	0.422–0.611
Indirect effect(b)	M	Y	0.123	0.008	14.514	0.000***	0.107–0.140
Direct effect (c')	X	Y	0.16	0.012	13.073	0.000***	0.136–0.184

Note: ***p < 0.001; B, unstandardized coefficient; SE, standard error; X, medication literacy; M, self-efficacy; Y, medication adherence.

In addition, the mediating effect test was conducted by the bootstrap method with 1000 samples. The results showed that the 95% confidence interval of the mediating effect value of self-efficacy did not include zero (95% CI: 0.051∼0.079, *Z* = 8.678, *p* < 0.001), indicating that self-efficacy had a significant mediating effect on the relationship between medication literacy and medication adherence.

The regression correlation coefficients of Path a, Path b, Path c and Path c' were all significant. Therefore, self-efficacy had a partial mediating effect on the relationship between medication literacy and medication adherence. Medication literacy predicted hypertensive patients’ adherence to medication partially through self-efficacy.

## Discussion

In this study, 60.9% of participating hypertensive patients were low adherent to their medication regimens. This result was consistent with findings in other studies worldwide (Warren-Findlow et al., 2012; Son and Won, 2017). Therefore, the majority of hypertensive patients in China and other countries have poor adherence to their medication regimens. That could be a major problem for hypertensive patients to reach an optimal blood pressure control. In addition, nonadherence to antihypertensive drugs could eventually accelerate the development of hypertension-related complications, increasing the hospital readmission rate and increasing the consumption of medical resources (Dragomir et al., 2010). Besides, education level, annual income, number of antihypertensive drugs prescribed and number of times antihypertensive drugs taken daily were identified as influencing factors of medication adherence in this study. Similar influencing factors of medication adherence for hypertensive patients have also been identified in previous studies (Al-Ruthia et al., 2017; Rampamba et al., 2018). The total score for the medication literacy scale was 23.83 ± 4.99 in our study. Several studies have also identified an insufficient medication literacy level using the same research tools as we did (Ma et al., 2019; Shi et al., 2019). Obviously, compared with the full score of 37, the medication literacy level for Chinese hypertensive patients need to be further improved. Inappropriate medication use was identified to be significantly associated with low medication literacy level (Chun-Hsien et al., 2017).

In addition, the results in the present study showed that education level, annual income, duration of hypertension, and number of antihypertensive drugs prescribed for hypertensive patients could also affect their medication literacy level. These findings were consistent with those in prior study (Ma et al., 2019). In previous study, occupational status and the type of medical insurance for hypertensive patients could also affect their medication literacy level (Ma et al., 2019). In the present study, patients with higher education level and annual income tended to have higher medication literacy and medication adherence levels. we speculate that patients with higher education and income might have more access to medication knowledge and have better understanding of antihypertensive drugs, which will be important basic abilities for patients to form positive attitudes and adherent behaviors to taking medication. Therefore, patients who are less educated and earned less should be targeted for medication literacy and medication adherence improvement. We also found that those who had longer duration of hypertension or had a smaller number of antihypertensive drugs prescribed were more likely to have higher medication literacy level. It indicated that health counsellors should focus on hypertensive patients who are with shorter duration since they were diagnosed and those who are prescribed with a more complexed medication regimen.

Furthermore, medication literacy was found to be positively correlated with medication adherence for hypertensive patients in the present study. The results of hierarchical regression analysis also showed that medication literacy was an independent predictor of medication adherence after controlling for sociodemographic and clinical information. This was consistent with the study of Shi et al. (Shi et al., 2019). The reason might be that patients with higher medication literacy are more likely to make medication decisions correctly according to acquired information. In contrast, inadequate medication literacy could result in misunderstanding of medication-related information or negative attitudes to taking antihypertensive drugs, leading to poor adherence to taking antihypertensive drugs.

Self-efficacy was found to be positively correlated with medication adherence for hypertensive patients. In addition, self-efficacy was also confirmed an independent predictor of medication adherence in the present study. This result was consistent with several previous studies (Bane and Mcelnay, 2010; Breaux-Shropshire et al., 2012; Francois, 2015) Individuals with a higher level of self-efficacy are more likely to be adherent to antihypertensive regimens. Possible reason might be that hypertensive patients who have insufficient self-efficacy negatively reckon they have no ability to persistent in lifetime medication taking.

After controlling for patient demographic and clinical characteristics, self-efficacy was found to be a partial mediator on the relationship between medication literacy and medication adherence in the present study. Medication literacy includes knowledge, attitude, behavior and skills to use specific medication for patients (Zhong et al., 2020). In the study of Shi et al. (Shi et al., 2019), knowledge, attitude, behavior and skills as well as the overall score of medication literacy were found to be significantly correlated with medication adherence, though, only attitude and behavior were confirmed as significant predictors of medication adherence. Moreover, identified significant predictors of attitude, behavior and annual income can only explain 15.8% of the variation in patients’ adherence level. However, in the present study, we found that medication literacy had a significant total effect on medication adherence after other variables including demographic and clinical characteristics were controlled. Therefore, medication literacy was verified as an independent predicator for medication adherence in hypertensive patients. Consequently, self-efficacy exerted a significant effect on partially mediating the association between medication literacy and medication adherence, and the mediating effect value was 28.7%. This result was consistent with a previous study, in which the mediating effect of self-efficacy on the association between health literacy and medication adherence among patients with diabetes was tested and confirmed (Huang et al., 2018). Despite optimal medication literacy including knowledge, attitude, behavior and skills in the process of antihypertensives administration was extremely important for patients to have a better adherence in taking antihypertensive drugs, self-efficacy also played a critical mediating role in promoting patients’ medication adherence. Possible explanation for this interaction is that optimal medication literacy could be basic essentials for hypertensive patients to process and administer antihypertensives in a correct and effective way, but higher self-efficacy even convinces themselves to believe that they have abilities to persist in taking antihypertensives in their lifetime. Basic essentials of optimal medication literacy level involve adequate hypertension related knowledge, positive attitudes to hypertension and treatment strategies, skills like numeracy and calculating, and correct behaviors in processing medication (Shi et al., 2019). In previous studies, self-efficacy has also been identified as an important mediating factor on the relationship among weight discrimination and depression with medication adherence (Richardson et al., 2014; Son and Won, 2017). Therefore, self-efficacy is a vital mediating predictor of medication adherence. It is imperative that self-efficacy should be targeted to address the medication adherence gap worldwide.

According to the results of this study, we can put forward some suggestions from two aspects to improve hypertensive patients’ adherence to their medication regimens. First, effective interventions to improve patients' medication literacy should be designed and implemented. In addition, hypertensive patients with suboptimal medication literacy should be tested using evaluation tools in the beginning. Besides, health education materials should be designed as simple and easy to understand as possible. Second, for hypertensive patients with low medication literacy, self-efficacy should also be focused on in order to promote their medication adherence. Some social cognitive and behavioral therapies in psychological treatment can be incorporated to improve self-efficacy for hypertension patients. For example, Sukwatjanee (Arissara, 2014) has effectively improved the perceived self-efficacy level of hypertension patients on a healthy diet by implementing a motivational project including health education, focus group discussion, diet supervision, mailed reminders and telephone consultation. Specifically, the knowledge gained through experience sharing, the understanding and self-confidence enhanced by group discussion, and the social support and authorization obtained by participating in incentive plans all played a significant role in the improvement of patients' self-efficacy.

## Study Limitations

There are some limitations to this study. First, self-reported tools were used to measure medication adherence in the present study. Adherence results obtained from objective measures such as automated pill counters or biochemical indicators might be more convincing. Second, although the *C-MLSHP* and *MASES-R* are validated and reliable scales to measure medication literacy and self-efficacy, they both lack cut-off points to classify specific levels. Finally, all variables in this cross-sectional study were collected in a questionnaire survey, so we were unable to determine the continuous changes in medication literacy, self-efficacy and medication adherence. Continuous-follow-up investigations should be carried out on patients with hypertension.

## Conclusion

Our study demonstrates that self-efficacy has a partial significant mediating effect on the relationship between medication literacy and medication adherence. Considering the prevalence of poor adherence to antihypertensive regimens among patients with hypertension, targeted interventions to improve patients’ self-efficacy could increase the confidence of hypertensive patients to adhere to their medication regimens. In addition, health care providers should be aware of the importance of medication literacy assessment and promotion in patients with hypertension.

## Data Availability Statement

The raw data supporting the conclusions of this article will be made available by the authors, without undue reservation.

## Ethics Statement

The studies involving human participants were reviewed and approved by the Ethics Committee of the Third Xiangya Hospital of Central South University (No. 2016-S050). The patients/participants provided their written informed consent to participate in this study.

## Author Contributions

ZZ was in charge of this whole project and designed and instructed the research; ZS made contributions to data analysis and drafted the manuscript; SS contributed to collecting data; SD instructed the data collection and data analysis.

## Funding

The program was supported by the National Natural Science Foundation of China (Project number: 71603290) and the Natural Science Foundation of Hunan Province, China (2018JJ2597).

## Conflict of Interest

The authors declare that the research was conducted in the absence of any commercial or financial relationships that could be construed as a potential conflict of interest.
